# 3D continuum phonon model for group-IV 2D materials

**DOI:** 10.3762/bjnano.8.136

**Published:** 2017-06-30

**Authors:** Morten Willatzen, Lok C Lew Yan Voon, Appala Naidu Gandi, Udo Schwingenschlögl

**Affiliations:** 1 Department of Photonics Engineering, Technical University of Denmark, Kgs. Lyngby, 2800, Denmark; 2 College of Science and Mathematics, The University of West Georgia, 1601 Maple St, Carrollton, GA 30117. USA; 3 PSE Division, King Abdullah University of Science and Technology, Thuwal 23955-6900, Kingdom of Saudi Arabia

**Keywords:** graphene, molybdenum disulfide, phonon, silicene, two-dimensional materials

## Abstract

A general three-dimensional continuum model of phonons in two-dimensional materials is developed. Our first-principles derivation includes full consideration of the lattice anisotropy and flexural modes perpendicular to the layers and can thus be applied to any two-dimensional material. In this paper, we use the model to not only compare the phonon spectra among the group-IV materials but also to study whether these phonons differ from those of a compound material such as molybdenum disulfide. The origin of quadratic modes is clarified. Mode coupling for both graphene and silicene is obtained, contrary to previous works. Our model allows us to predict the existence of confined optical phonon modes for the group-IV materials but not for molybdenum disulfide. A comparison of the long-wavelength modes to density-functional results is included.

## Introduction

Phonon spectra in two-dimensional (2D) nanomaterials have almost exclusively been computed using density-functional theory (DFT) based codes. One of the earliest applications to group-IV elemental 2D materials was for the important prediction of the stability of silicene and germanene [1]. These are complex calculations and prone to qualitative errors due to the various approximations such as convergence criteria and use of approximate functionals [2]. Even the stability or not of a given structure could be incorrectly inferred. For example, borophene and indiene have been predicted to be unstable in one paper [3], though other calculations (and in the case of borophene, even experiments [4]) have obtained opposite results [5–6]. In addition to obtaining a spectrum, it is often also useful to be able to predict and/or interpret properties of the spectrum based upon either microscopic or symmetric arguments. An excellent example is the prediction of a Dirac cone for silicene on the basis of symmetry [7] when DFT calculations either failed to recognize it [8] or were unable to explain it [1].

An alternative model of lattice vibrations is a classical continuum model, which is expected to reproduce most accurately phonons with wavelengths longer than lattice separations, i.e., near *k* = 0. One of the earliest such models applied to an ionic crystal slab is that of Fuchs and Kliewer [9], from which they deduced that transverse optical (TO) and longitudinal optical (LO) modes have different frequencies at *k* ≈ 0 as well as the existence of surface optical (SO) modes with an exponential dependence away from the slab. Slightly different models have been introduced by a number of authors for graphene [10–13]. One commonality is to treat the sheet as strictly two-dimensional. Additionally, instead of deriving the phonon dispersion relations from first principles, they all assumed the known results that there are in-plane acoustic modes with linear dispersions and out-of-plane transverse acoustic modes with quadratic dispersions (the latter being consistent with the elastic theory of thin plates) to construct either a Lagrangian [10] or equations of motion [11]. Goupalov also considered optical phonons but simply parameterized the dispersion relations to match the experimental data. In all of the above, the out-of-plane vibrations were assumed decoupled from the in-plane ones.

In this paper, a continuum theory of acoustic and optical phonons in 2D nanomaterials is derived from first principles, contrary to earlier approaches, by starting with the elastic and electric equations, and taking into account the full crystalline symmetry and piezoelectric couplings when allowed by symmetry. We apply the theory to obtain the phonons in group-IV elemental 2D materials. Given that there are two fundamental structures for the free-standing sheets (the flat hexagonal structure of graphene and the buckled hexagonal structure of the other elements – we will refer to silicene as the prototypical example of the latter), we will consider both of them. It should be recognized that, to date, silicene has been grown on substrates in different reconstruction state. The reconstruction is an atomic-scale distinction that is not describable by the current continuum model. Substrate effects on the phonons can be considered in an extended model that would need to be solved numerically. This can be studied in a future publication as it is beyond the analytical solutions sought for in the current manuscript. Furthermore, we have included a study of a well-known compound 2D material (molybdenum disulfide MoS_2_) in order to further understand the properties derived for the elemental materials.

## Results and Discussion

### Continuum model

The 2D materials will be treated as 3D thin-plate materials in the following, well aware that the out-of-plane dimension contains one or a few atomic layers. In general, this would allow one to study multilayers though we will only focus on monolayers in this paper. Nonetheless, it is important to keep the third coordinate in the analysis to reveal the true phonon dispersions as observed experimentally and in DFT calculations.

The general 3D elastic equations are given by the equation of motion [14]

[1]
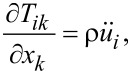


where *T_ik_* is the stress tensor, ρ is the mass density, and *u_i_* is the displacement. Equation 1 contains all the physics of the problem. In order to simplify and then solve it, it is necessary to specify the crystal symmetry of the vibrating system.

The three problems considered in this article are graphene, silicene and MoS_2_. The Bravais lattice symmetries of the single-layer graphene (*D_6h_* ≡ 6/*mmm*) and MoS_2_ (
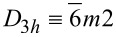
) structures belong to the hexagonal system, while silicene belongs to the trigonal system (point group *D*_3_*_d_*). Graphene and silicene are non-piezoelectric materials because of the inversion symmetry of the unit cell, while MoS_2_ is piezoelectric because its unit cell exhibits inversion asymmetry.

### Application: graphene

For both graphene and MoS_2_, the general form of the stiffness tensor for hexagonal structures is

[2]
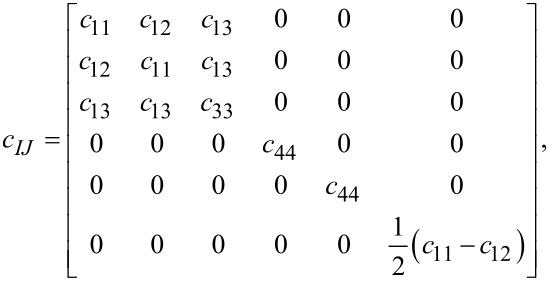


and the stress–strain relations *T_I_* = *c_IJ_S_J_* for graphene become

[3]
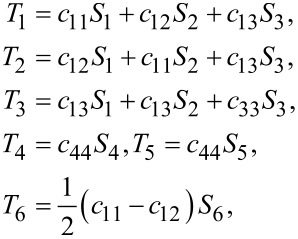


where *T_I_* and *S_J_* denote stress and strain, respectively. Here we have used Voigt notation for tensors. The latter equations are different for MoS_2_ due to the presence of piezoelecticity.

#### Elastic equations

Combining Equation 1 and Equation 3, we obtain for graphene

[4]
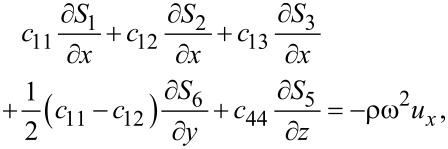


[5]
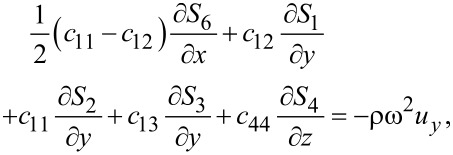


[6]
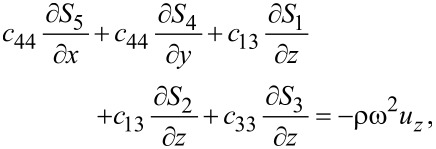


where ω is the vibrational (angular) frequency. The displacements *u_x_* and *u_y_* are in-plane displacements, and *u_z_* is the out-of-plane displacement.

Expressing the latter set of equations in the displacements, we get

[7]
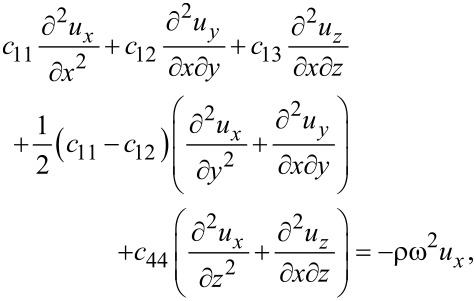


[8]
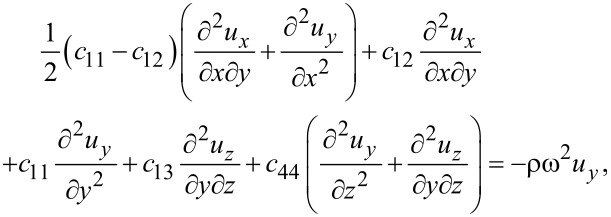


[9]
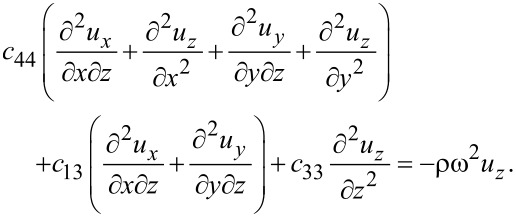


A solution of the combined system (Equation 7–Equation 9) with appropriate boundary conditions allows for the determination of the displacements *u_x_*, *u_y_*, *u_z_*. Note that the displacements in the three directions are coupled. This is different from the 2D model assumed before, which led to a decoupling of the out-of-plane vibrations. The coupling is a consequence of the finite thickness of the sheet with no mirror symmetry imposed. Thus, our model is sufficiently general to apply to multilayers. Earlier DFT calculations [15] had argued that there is no coupling between the in-plane and out-of-plane modes due to the mirror symmetry of graphene, leading to fewer scattering channels and, therefore, a higher thermal conductivity compared to, e.g., silicene. Our model reveals that such mode coupling, even when present, would occur for large *k_z_* values (due to the small thicknesses) and, hence, would be unlikely to have a significant impact.

#### Acoustic phonons

The phonons are normal mode solutions to the equations of motion. To proceed from the graphene elastic equations, we make the following plane wave ansatz

[10]
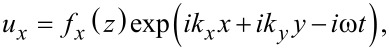


[11]
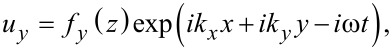


[12]
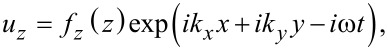


where *k_x_* and *k_y_* are wave numbers. Inserting Equation 10–Equation 12 into Equation 7–Equation 9 yields the following matrix expression in the unknown functions *f_x_*, *f_y_*, and *f_z_*:

[13]
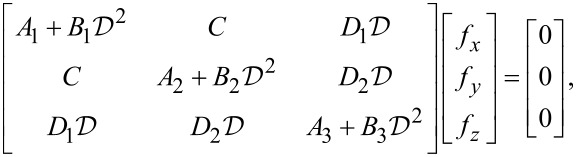


where 
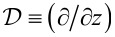
, and

[14]
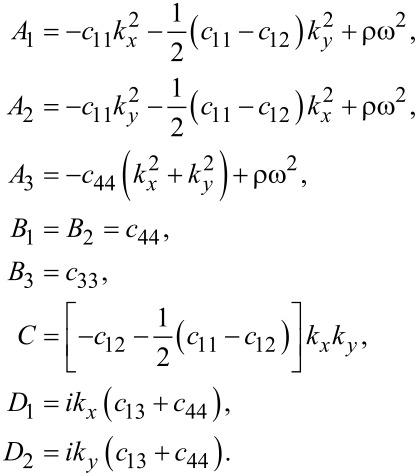


A semi-analytic solution can be easily obtained for the case when *k_y_* = 0. In this case, *C* = *D*_2_ = 0 and the *u_y_* displacement decouples from the displacements *u_x_* and *u_z_*. It follows that *f_y_* obeys the wave equation

[15]
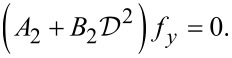


The solution to this differential equation can be found immediately by imposing the vacuum boundary condition

[16]
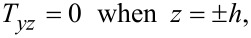


i.e.,

[17]
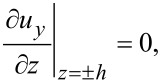


where *2h* is the graphene layer thickness and −*h*,*h* define the graphene layer boundaries.

We obtain

[18]
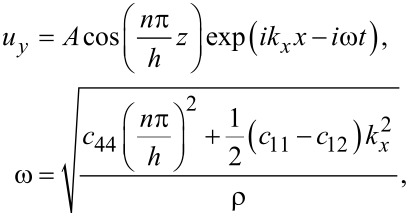


where *n* = 0,1,2,3,…

For the coupled system *f_x_*–*f_z_*, the determinantal system obtained from Equation 13 leads to the solutions

[19]
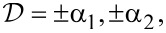


where

[20]
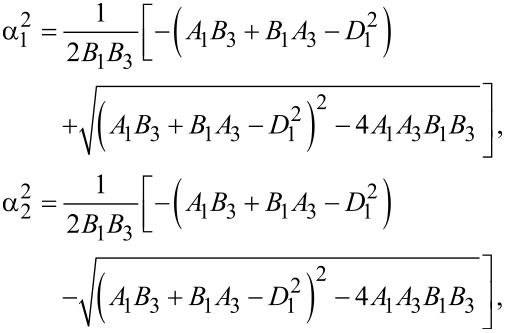


and

[21]
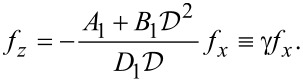


Let us introduce the notation {α_1_,α_2_,α_3_,α_4_} ≡ {α_1_,−α_1_,α_2_,−α_2_} and

[22]
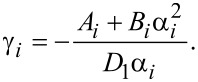


Then we have

[23]
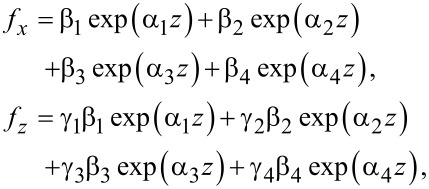


where β*_i_* are unknown coefficients.

A 4 × 4 matrix equation in β*_i_* is finally obtained by invoking the mechanical boundary conditions (graphene in vacuum)

[24]
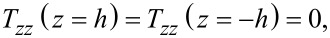


[25]
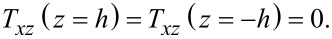


Observe that

[26]
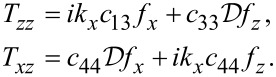


Solving the determinantal equation for the latter 4 × 4 matrix equation as a function of *k_x_* specifies a discrete set of (band) eigenfrequencies ω*_i_*(*k_x_*) and the corresponding eigenmodes *f_x_*, *f_z_* where *i* denotes the band index. A numerical solution reveals the out-of-plane mode to be quadratic in nature.

#### Surface optical phonons

Surface optical phonons can be derived by solving for the electrostatic potential via the Maxwell–Poisson equation for the displacement field *D_i_*.

For graphene,

[27]
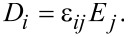


Using the fact that the dielectric function is given by

[28]
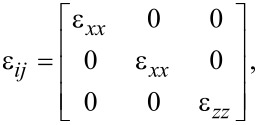


we find

[29]
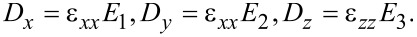


The Maxwell–Poisson equation in the absence of free charges reads

[30]
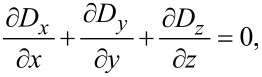


and finally becomes

[31]
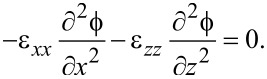


The solution in the graphene slab becomes

[32]
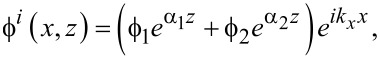


where

[33]
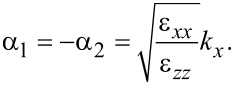


This gives for the electric field components

[34]
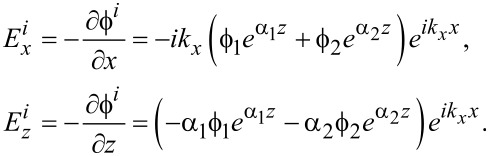


Continuity in *E_x_* at the slab interfaces with vacuum at *z* = ±*h* requires

[35]
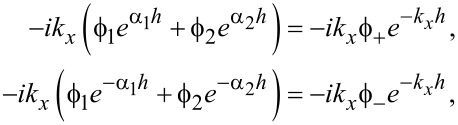


and, similarly, continuity in the normal electric displacement requires

[36]
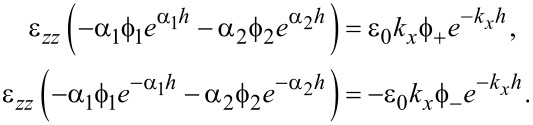


Solving the secular equation in 
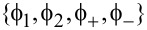
 leads to


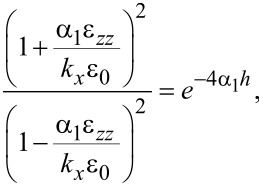


which is equivalent to

[37]
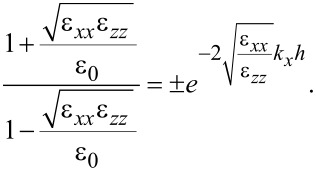


We note in passing that the latter equation agrees with the result of Licari and Evrard [16] for interface optical phonon modes in cubic crystals where symmetry forces ε*_xx_* = ε*_zz_* ≡ ε. In the cubic case, however, confined optical phonon modes also exist at the LO phonon frequency since ε(ω_LO_) = 0.

#### Confined optical phonons

Confined optical phonons are also obtained by starting with the Maxwell–Poisson equation (Equation 31). The transverse electric field and normal electric displacement are

[38]
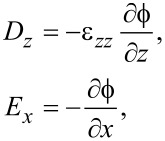


neglecting the DC component (static polarization) to the electric displacement. Confinement of the optical phonon modes implies 
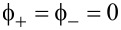
. Assuming [type I]

[39]
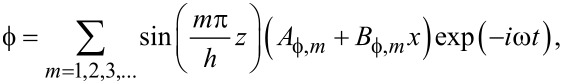


where 
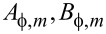
 are constants, the Maxwell–Poisson equation and the boundary conditions are fulfilled if ε*_zz_*(ω) = 0. Hence confined optical phonons exist in graphene.

### Application: silicene

We refer to silicene as the canonical example of the other group-IV materials even though the following derivation and qualitative properties apply to all of them since they all have the same symmetry of the buckled hexagonal structure, leading to the trigonal *D*_3_*_d_* point group.

The general form of the stiffness tensor for *D*_3_*_d_* trigonal structures is

[40]
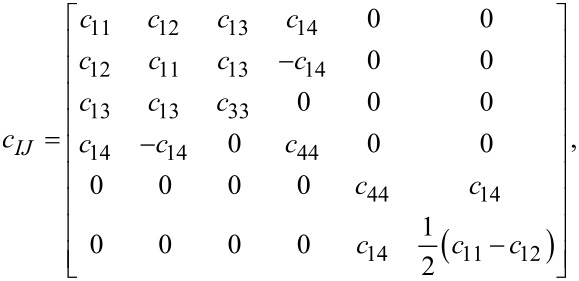


and the stress–strain relations become

[41]
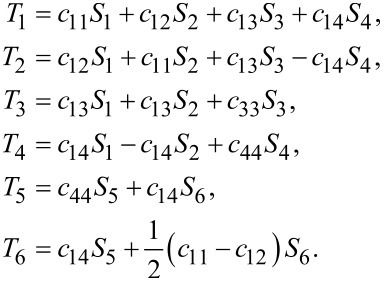


The only difference compared to the corresponding equations for graphene are the terms containing *c*_14_.

#### Elastic equations

The elastic equations for silicene then read

[42]
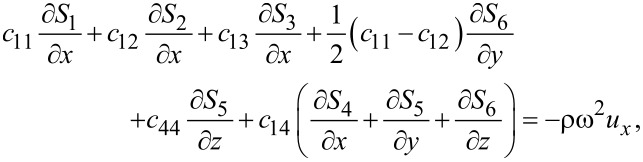


[43]
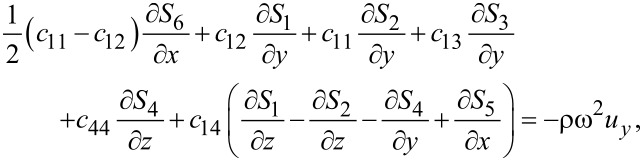


[44]
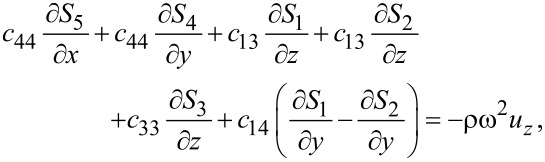


or, in terms of the displacements,

[45]
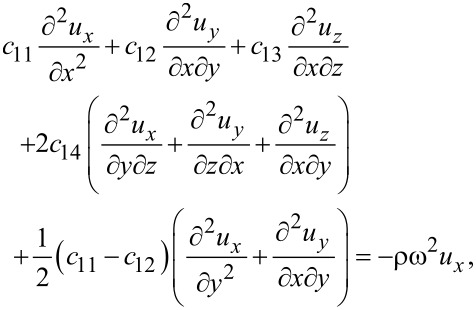


[46]
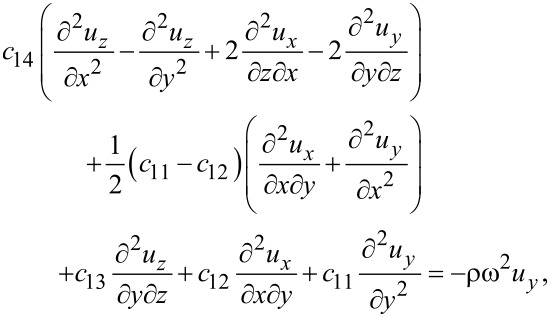


[47]
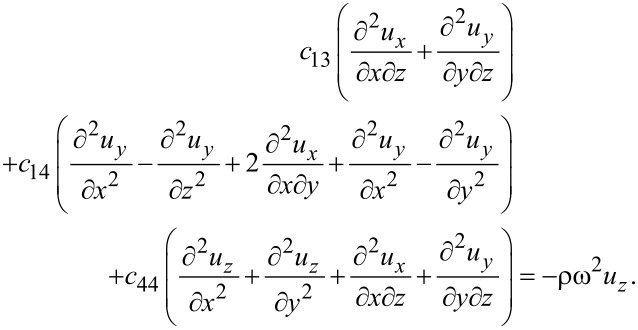


Again, the differences compared to graphene are the *c*_14_ terms. The electric equation for trigonal structures is exactly the same as for hexagonal structures since the permittivity matrix is the same. Hence, the general method for finding surface optical phonon modes repeats the description of graphene. Qualitatively, there is therefore no difference among the phonons for all the group-IV elemental 2D materials.

### Application: MoS_2_

While the primary focus of this paper is on the properties of phonons of the group-IV elemental materials, it is important to know if there are properties of the phonons that are due to these being elemental. Hence, we will now consider the phonons for MoS_2_ as a prototypical compound 2D material because of its extensively studied properties. As already mentioned, the lack of inversion symmetry leads to the new phenomenon of piezoelectricity.

#### Elastic and electric equations for MoS_2_

The stiffness tensor for MoS_2_ is the same as for graphene, Equation 2, because of the same hexagonal symmetry. However, the stress–strain relations are different because of the presence of piezoelectricity. Specifically, there are additional contributions to the stress–strain constitutive relations:

[48]
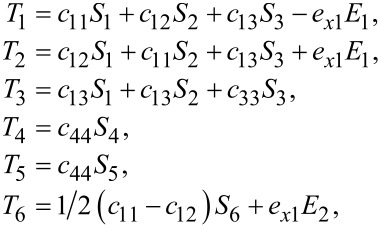


by use of the piezoelectric *e*-tensor:

[49]
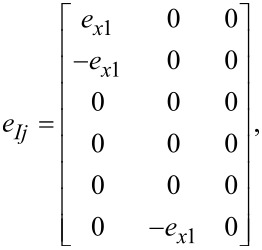


and the equation

[50]
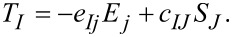


In the above, *E_j_* is the internal electric field.

With the above result for the stress tensor including piezoelectric contributions, we find the elastic equations for MoS_2_

[51]
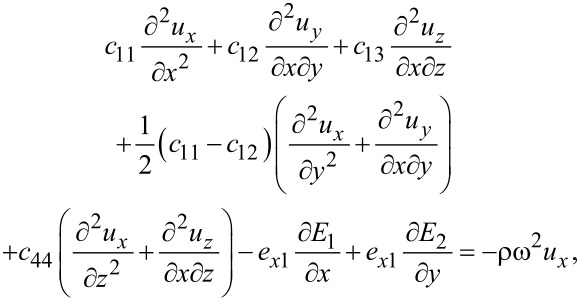


[52]
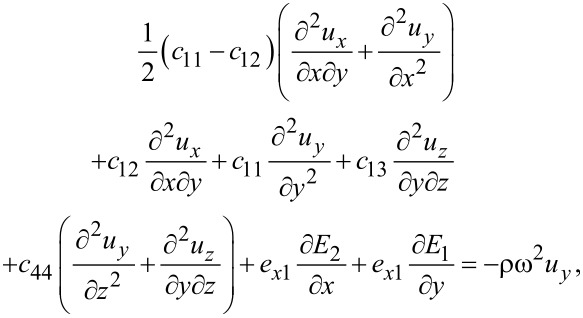


[53]
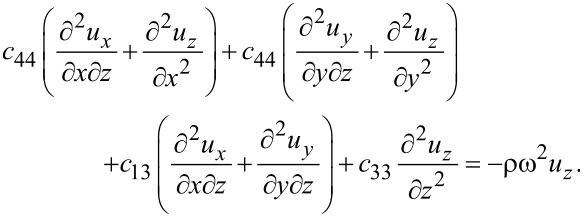


This system of equations is only complete if we also solve for the Maxwell–Poisson equation. For MoS_2_, the displacement field *D_i_* is

[54]
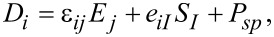


with *P_sp_* being the spontaneous polarization contribution. The dielectric function is the same as for graphene (Equation 28) and we find

[55]
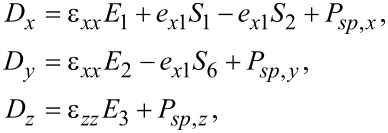


where *P_sp,x_*, *P_sp,y_* and *P_sp,z_* denote the spontaneous polarization components along the *x*-, *x*- and *z*-direction, respectively. The Maxwell–Poisson equation (Equation 30) becomes

[56]
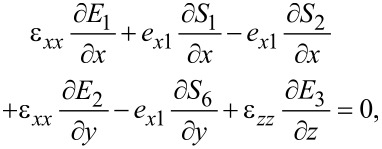


because the spontaneous polarization *P_sp_* is constant in space. A solution of the combined system Equation 51–Equation 53 and Equation 56 with appropriate boundary conditions allows for the determination of the electric field (or electric potential 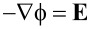) and the displacements *u_x_*, *u_y_* and *u_z_*.

#### Phonon modes

In the case of MoS_2_, the general solutions are more complicated. One can still make the plane wave ansatz,

[57]
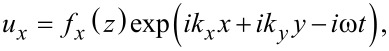


[58]
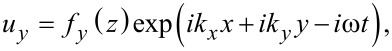


[59]
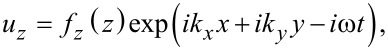


[60]
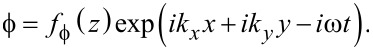


Now, inserting Equation 57–Equation 60 in Equation 51–Equation 53 and Equation 56 yields the 4 × 4 matrix expression in the unknown functions *f_x_*, *f_y_*, *f_z_* and 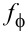


[61]
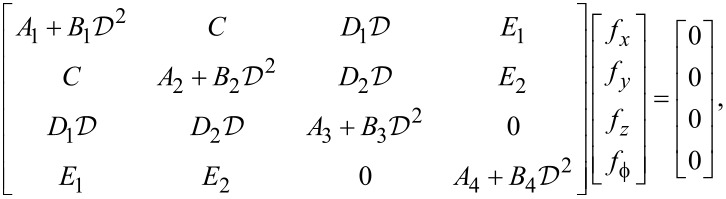


where

[62]
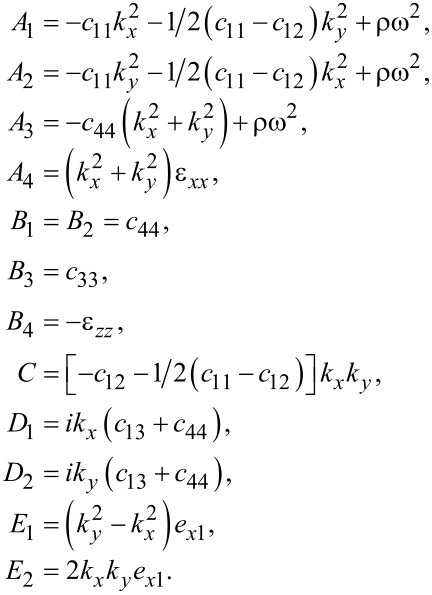


For the case, *k_y_* = 0, we have *C* = *D*_2_ = *E*_2_ = 0 and *u_y_* decouples from *u_x_*, *u_z_* and 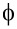. The wave equation for the *u_y_* mode is the same as for graphene. To determine the general solution for the other displacement components, we once again solve the determinantal equation for the 3 × 3 sub-matrix in the components *f_x_*, *f_z_* and 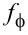 . The result is

[63]
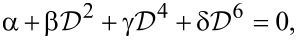


where

[64]
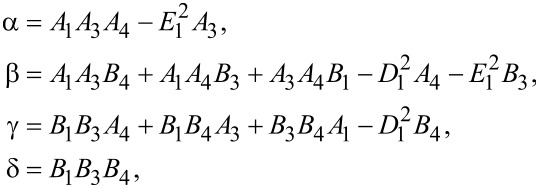


and six roots (three pairs of opposite signs) exist. It also follows that

[65]
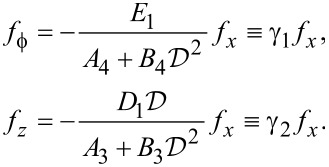


Hence the general solution is, using a notation similar to the case of graphene,

[66]
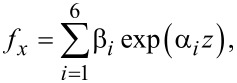


[67]
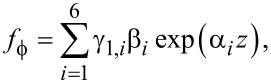


[68]
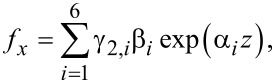


where β*_i_* are unknown coefficients.

The Maxwell–Poisson equation in vacuum (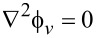) has the general solution

[69]
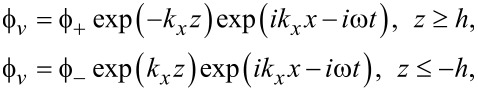


where 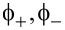 are unknown constants. An 8 × 8 matrix equation in β*_i_*, 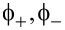 is finally obtained by invoking the four mechanical boundary conditions at *z* = *h*,*−h*

[70]
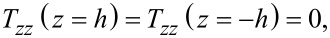


[71]
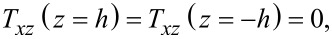


and the four electric boundary conditions at *z* = *h*,*−h*,

[72]
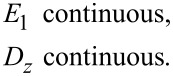


The mechanical boundary conditions are the same as for graphene,

[73]
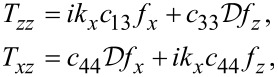


but the electric boundary conditions are (written out)

[74]
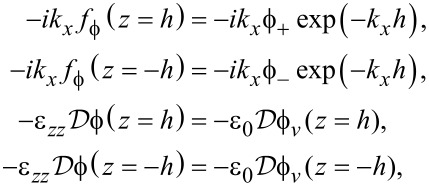


where ε_0_ is the vacuum permittivity. Solving the determinantal equation for the 8 × 8 matrix equation as a function of *k_x_* specifies a discrete set of (band) eigenfrequencies ω*_i_*(*k_x_*) and the corresponding eigenmodes *f_x_*, *f_z_* and 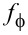, where *i* denotes the band index.

#### Confined phonon modes

An important result concerning confined optical phonons can be obtained without solving the determinantal equations. From the elastic equations (Equation 51 and Equation 53) and the electric equation (Equation 56) we have, assuming *k_y_* = 0,

[75]
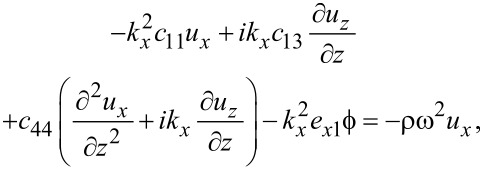


[76]
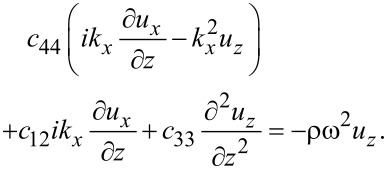


[77]
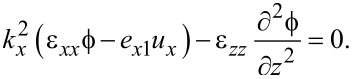


Inspection of the above set of equations reveals that confined phonon solutions can be sought in the form [type I]

[78]
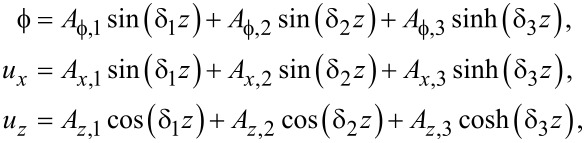


or in the form [type II]

[79]
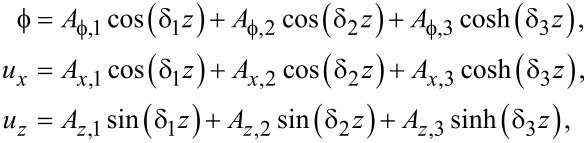


apart from a multiplying factor exp(*ik_x_x* − *i*ω*t*). The above choice reflects an expectation that four roots are real (two pairs of opposite-signed roots) and two imaginary (one pair of opposite-signed roots).

From Equation 75–Equation 77 follows

[80]
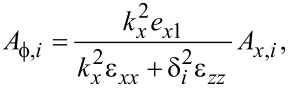


[81]
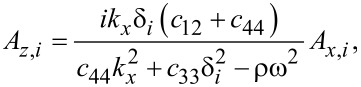


[82]
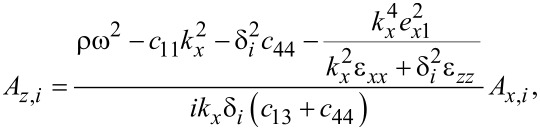


where *i* = 1,2,3. Note that a maximum of two of the latter relations can be independent since the values of δ*_i_* are found by setting the system determinant to zero.

A general confined optical phonon mode can now be written as a Fourier series expansion [type I]

[83]
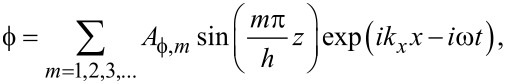


[84]
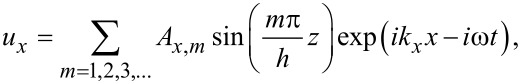


[85]
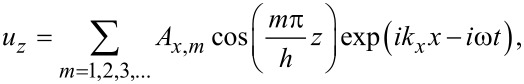


since this construction implies *E_x_*(*z* = ±*h*) = 0 and 
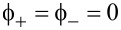
 using continuity in the transverse electric field at the interfaces. Equation 75–Equation 77 must still apply, however, in particular combining Equation 81–Equation 82 demands

[86]
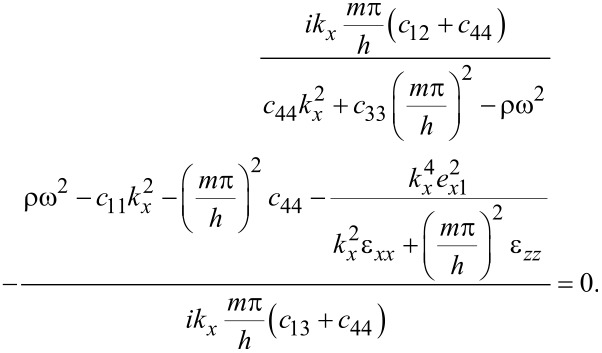


For each term *m*, this latter condition, in general, would lead to a different ω*_m_*. However, a normal mode is characterized by a unique frequency ω. Hence, for confined optical phonon modes, only one term in *m* is allowed in the general Fourier series expansions above. Further, imposing continuity in the normal electric displacement component at *z* = ±*h* by use of the third equation in Equation 55 gives

[87]
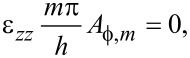


and 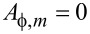 unless accidentally ε*_zz_*(ω*_m_*) = 0 (treated separately in the next paragraph). For ε*_zz_*(ω*_m_*) ≠ 0, it follows from Equation 80 that *A_x,m_* = 0 and Equation 81 yields *A_z,m_* = 0. The conclusion is that confined optical phonons in the general case cannot exist in MoS_2_! Note that for graphene, since it is a non-piezoelectric material, confined optical phonon modes do exist. The difference between the two materials lies in Equation 77, specifically in the term containing the piezoelectric coefficient.

If ε*_zz_* = 0, continuity of the normal electric displacement component requires, as before, 
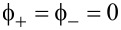
 and we return to the situation treated in the previous subsection about confined optical phonons. We need to consider two possible cases: (a) ε*_xx_*(ω) = 0 when ε*_zz_*(ω) = 0 and (b) ε*_xx_*(ω) ≠ 0 when ε*_zz_*(ω) = 0. In case (a), we immediately obtain 
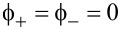
 from continuity in the normal electric displacement. Further, Equation 77 yields *u_x_* = 0 everywhere as a function of *z*. Equation 83–Equation 85 must hold from continuity in the transverse electric field. Finally, Equation 75 gives

[88]
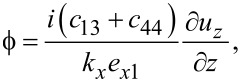


and Equation 76 requires [type I]

[89]
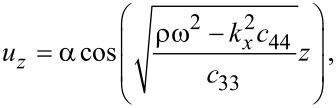


and the condition

[90]
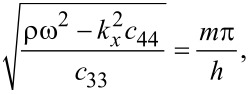


which is not fulfilled, unless accidental degeneracy applies, simultaneous with ε*_xx_*(ω) = ε*_zz_*(ω) = 0. In case (b), Equation 86 can be used and, unless accidental degeneracy applies, the obtained ω*_m_* values do not fulfill ε*_zz_*(ω) = 0. In conclusion, confined optical phonon modes do not exist in the case ε*_zz_* = 0.

### Computed dispersion relations

In order to illustrate the validity of the phonon dispersion relations obtained from our model, we compare them to DFT calculations. The continuum theory will require as input elasticity constants, piezoelectric coefficients, and dielectric functions.

#### DFT

**Figure 1:**
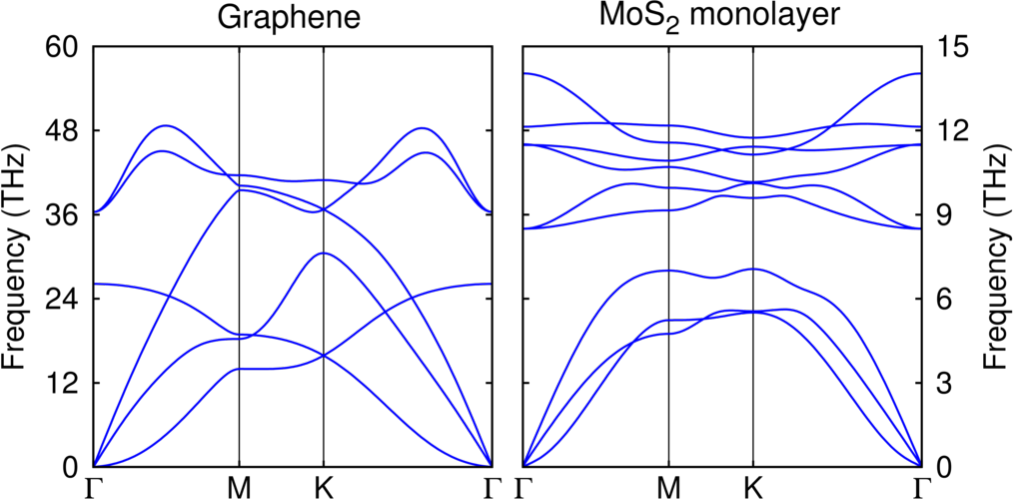
Dispersion relations for the phonon modes of single-layer graphene (left) and MoS_2_ (right), from DFT calculations.

We first give the standard phonon dispersion relation as obtained from DFT calculations (Figure 1). They are obtained from first principles calculations using the Vienna ab initio simulation package (VASP) [17] with a kinetic energy cut-off of 500 eV in the expansion of the electronic wave functions. Four C and six Mo and S valence electrons are considered. The generalized gradient approximation of the exchange–correlation potential in the Perdew–Burke–Ernzerhof flavor is employed [18]. Brillouin-zone integrations are performed using the tetrahedron method with Blöchl corrections [19]. We construct cells consisting of the monolayer and an approximately 15 Å thick vacuum region along the *c* direction. Structure optimizations are performed on Γ-centered 24 × 24 × 1 *k*-meshes. A direct method based on 4 × 4 × 1 supercells is used for obtaining the phonon dispersions within the harmonic approximation [20]. Forces are evaluated on 3 × 3 × 1 *k*-meshes, including long-range dipole contributions to the dynamical matrix following the method of [21]. Born effective charges and dielectric tensors are obtained within perturbation theory [22] and elastic constants by the homogeneous deformation method [23]. The elastic stiffness tensor and frequency-dependent dielectric tensor (independent particle approximation [24]) are calculated on Γ-centered 36 × 36 × 1 *k*-meshes.

#### Continuum model: graphene

The following parameters are found using DFT calculations as explained above: *c*_11_*d* = 345 Pa·m, *c*_12_*d* = 73 Pa·m, *c*_13_*d* = 0.00387 Pa·m, *c*_33_*d* = 0.531 Pa·m, *c*_44_*d* = 0.0535 Pa·m, *c*_66_*d* = 136 Pa·m, *d* = 3.4·10^−8^ m, and ρ_2D_ = 7.61·10^−7^ kg/m^2^. For the permittivity data we used the DC values ε*_xx_* = 4.4ε_0_ and ε*_zz_* = 1.3ε_0_.

**Figure 2:**
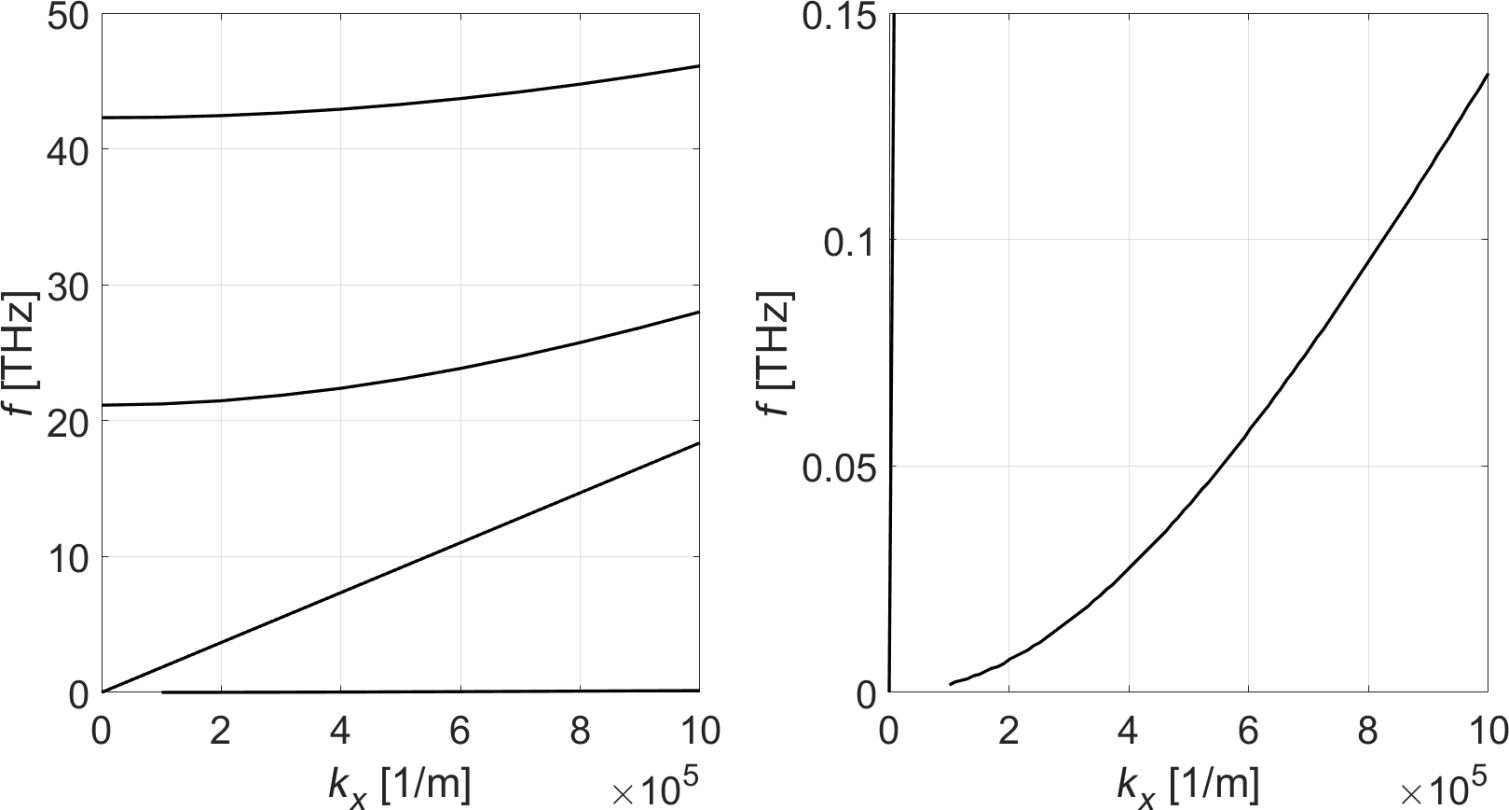
Dispersion relations for the coupled *u_x_*–*u_z_* acoustic phonon modes of single-layer graphene. The right plot is a zoomed version of the left plot. Note the nonlinear dispersion of one acoustic mode away from the Γ point. There is also one acoustic mode showing linear dispersion.

**Figure 3:**
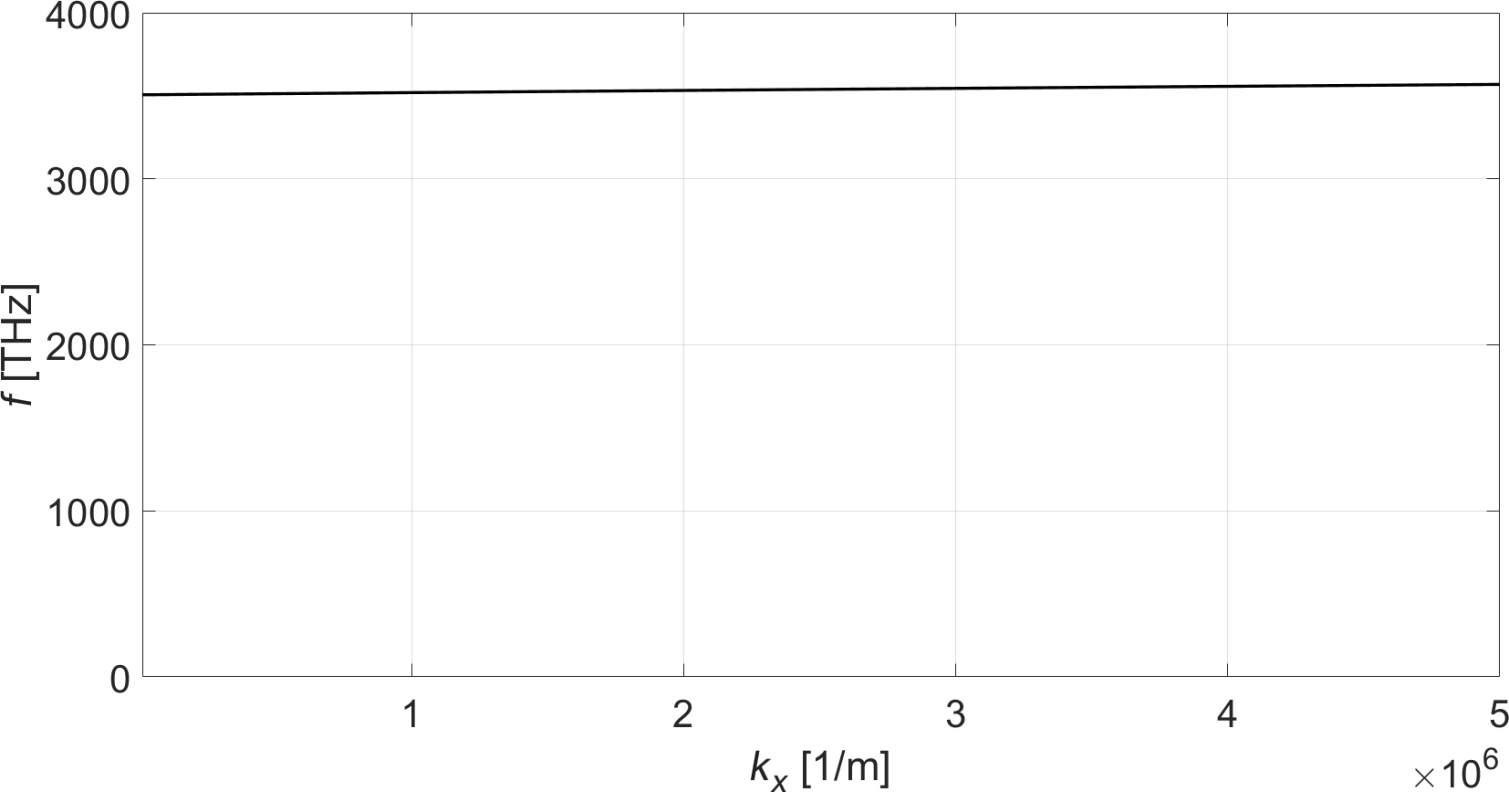
Dispersion relation for the optical phonon modes of single-layer graphene.

In Figure 2, we show the frequency vs wavenumber (ω–*k_x_*) dispersion in the vicinity of the Γ point (*k_y_* = 0). Evidently, one mode shows a parabolic dispersion and one mode is linear. There are two higher-order modes originating from the boundary conditions along the *z* coordinate. The dispersion curves associated with the *u_y_* vibrations decoupled from the *u_x_* − *u_z_* vibrations are not shown in the plots. We emphasize that for graphene optical and acoustic phonon modes decouple and are computed separately as described above. In Figure 3, the dispersion curves for optical phonon modes near the Γ point are shown.

#### Continuum model: MoS_2_

**Figure 4:**
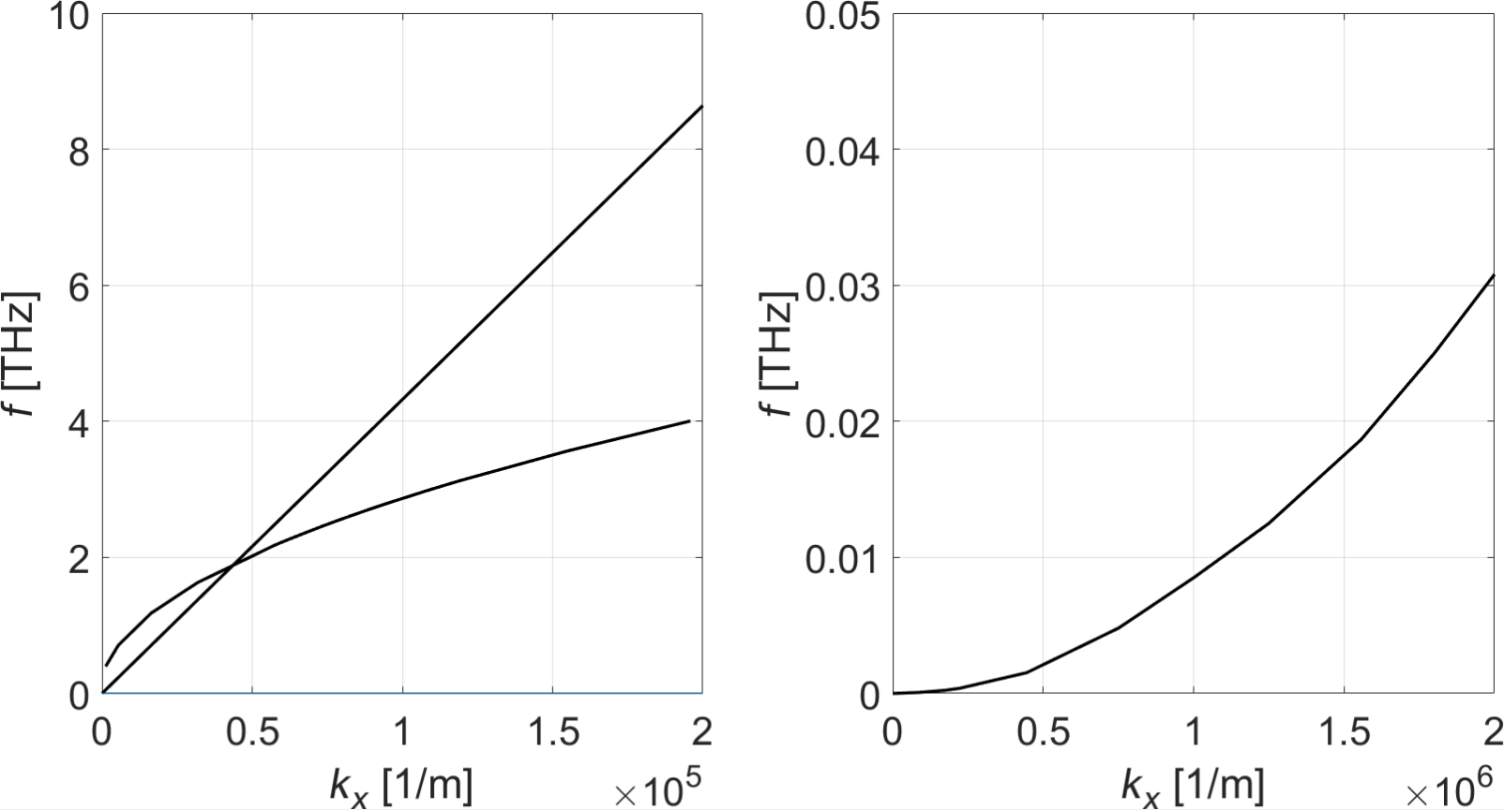
Dispersion relations for the coupled *u_x_*–*u_z_*–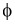 phonon modes of single-layer MoS_2_. The right plot is a zoomed version of the left plot. Note again the parabolic dispersion of one predominantly acoustic mode away from the Γ point besides a linear dispersion predominant acoustic mode. We also obtain a second nonlinear mode starting at (*k*,ω) = (0,0), which stems from the rather complicated frequency-dependent permittivity of MoS_2_.

For single-layer MoS_2_, we use the following parameters: *c*_11_*d* = 140 Pa·m, *c*_12_*d* = 33 Pa·m, *c*_13_d*=*-0.013 Pa·m, *c*_33_*d* = 0.078 Pa·m, *c*_44_*d* = −1.07 Pa·m, *c*_66_*d* = 53.7 Pa·m, *d* = 6.2·10^−10^ m, ρ_2D_ = 3.1 ·10^−6^ kg/m^2^, *e_x_*_1_ = 0.5 C/m^2^. These elasticity parameters were again computed using VASP. The frequency-dependent permittivity values are taken from [25] and the piezoelectric constant *e_x_*_1_ is from Ref. [26]. In the case of MoS_2_, all optical and acoustic phonon modes are computed by solving the combined set of elastic and electric equations and associated slab boundary conditions as described above. We note in passing that both confined and surface acousto-optical phonon modes are found using the present formalism. In Figure 4, we show the frequency vs wavenumber (ω–*k_x_*) dispersion in the vicinity of the Γ point (*k_y_* = 0).

## Conclusion

A three-dimensional, first-principles, continuum elasticity theory was developed for two-dimensional materials. Piezoelectric materials required the simultaneous consideration of the electrostatics equations. Application to graphene, silicene and MoS_2_ revealed a number of interesting results. The out-of-plane vibrations were coupled to the in-plane motion for graphene (and for silicene as well), contrary to previous results using infinitely thin sheets. Acoustic modes with linear and quadratic dispersions were obtained, in agreement with experimental results and other models. We predict the existence of confined optical modes in all of the elemental group-IV materials and the nonexistence of confined optical modes in MoS_2_. Our model will be applied to other 2D materials as well as to multilayers in future work. Additionally, the results of this model can be combined with the solution to the electron problem to compute electron–phonon scattering rates. The latter would be useful for understanding a number of physical properties as well as for applications.
